# A randomized controlled trial to address a multimodal intervention in the elderly: the effects of the CAMINN study

**DOI:** 10.1007/s40520-026-03371-x

**Published:** 2026-03-25

**Authors:** Guillermo Palacios-Navarro, Pedro Ramos, David del Río, Santiago Gascón-Santos

**Affiliations:** 1 Department of Electronic Engineering and Communications, University of Zaragoza, Teruel, Spain; 2 Department of Innovation, Ingesan-OHLA, Madrid, Spain; 3 Department of Psychology and Sociology, University of Zaragoza, Teruel, Spain

**Keywords:** Aging, Cognition, Community-dwelling, Neuropsychology, Quality of life

## Abstract

**Background:**

Older adults face multiple challenges that may negatively affect quality of life, including physical decline, dependency, social isolation, and reduced emotional support. These challenges affect both community-dwelling individuals and those living in nursing homes. This study explores the potential benefits of a multicomponent video call intervention targeting multiple biopsychosocial domains in older adults delivered remotely via the VERA system.

**Methods:**

A pragmatic, exploratory randomized controlled trial was conducted with 187 participants aged 56 to 101 years, allocated to a control group and an experimental group. Only the experimental group received a multicomponent intervention delivered via video calls over 18 months. Outcomes were assessed at baseline, 9 months, and 18 months across eight domains: cognition, quality of life, depression, perceived loneliness, basic and instrumental activities of daily living, balance, and gait. Standardized instruments were used, including the Mini Mental State Examination (MMSE), FUMAT scale, the abbreviated version of the Yesavage Geriatric Depression Scale (GDS), the revised ESTE scale, the Barthel’s scale, the Lawton and Brody’s test and the Tineti’s scale. Longitudinal changes were analyzed using linear mixed-effects models.

**Results:**

Favorable longitudinal changes were observed in the experimental group for cognitive functioning, depressive symptoms, perceived loneliness, quality of life, and basic activities of daily living. Significant group × time interactions indicated differential trajectories compared with the control group. In contrast, no meaningful intervention-related changes were detected for instrumental activities of daily living or balance and gait.

**Conclusions:**

The intervention is associated with positive longitudinal changes in several psychosocial and basic functional domains in older adults living at home or in residential care settings. While the findings suggest potential clinical relevance, they should be interpreted within an exploratory framework, and further research is needed to confirm effectiveness, sustainability, and generalizability. The trial was registered on ClinicalTrials.gov (Identifier: NCT07362381).

**Supplementary Information:**

The online version contains supplementary material available at 10.1007/s40520-026-03371-x.

## Introduction

The aging population faces numerous challenges that impact their quality of life (QoL), whether they reside in long-term care facilities or remain in their own homes. One of the most pressing issues is the decline in physical health, often associated with chronic conditions such as cardiovascular disease, diabetes, and neurodegenerative disorders [1]. This deterioration frequently leads to increased dependency and reduced autonomy [2].

In care homes, older adults often face difficulties stemming from staff shortages, impersonal care, and a lack of emotional support [3]. Furthermore, the rigid structure of many of these institutions can lead to feelings of loss of autonomy and alienation from family and community. In contrast, those who remain in their own homes may have difficulty accessing healthcare services, adapting their homes, or obtaining sufficient social support, especially when living alone or in rural areas [4].

Aging entails not only physical decline, but also changes in cognitive, emotional, social, and functional domains [5]. Mental health concerns are common in both contexts. Depression, anxiety, and cognitive decline are exacerbated by loneliness and social isolation, which are prevalent regardless of the living arrangement [6]. In this regard, there is evidence showing how belonging to social groups (such as support groups or group therapy) improves mental health, reduces depressive symptoms, and protects against isolation [7]. The study of Casañas-Sánchez et al. [8] evaluated the effectiveness of a psychoeducational group intervention addressing depression from multiple angles, including biological, psychological, and social aspects, with a significant reduction in anxiety and depression symptoms, as well as a decrease in the number of medical visits.

In recent decades, loneliness has received increasing attention as a significant risk factor for adverse health outcomes, including increased mortality risk [9], reduced self-care behaviors, and poorer adherence to prescribed treatments [10]. A recent systematic review conducted by Puyané et al. [11] highlighted the high prevalence of loneliness and social isolation among older adults living in the community, identifying key risk factors such as declining health, widowhood, and the erosion of social networks. The study of O’Súilleabháin et al. [12] revealed an 18.6% increase in overall mortality associated with emotional loneliness after a 19-year follow-up of patients. The impacts of loneliness and isolation are far-reaching, contributing to a heightened risk of both physical and mental health issues, including cardiovascular disease, anxiety, depression, and cognitive decline. On the other hand, depression in the geriatric population often follows a chronic and recurrent course. It is closely linked to biopsychosocial factors such as loneliness, reduced support networks, concomitant medical illnesses, side effects of pharmacological treatments, and family-related stressors [13]. Factors such as loss of relationships, sensory impairment, stressful events, role changes, and perceived loneliness can precipitate depressive episodes in old age [14].

The literature reviewed includes many interventions and programs focused directly on a single psychosocial aspect in community dwelling older people, such as perceived loneliness [15], depression [16, 17] or quality of life [18]. The former study was based on a personalized residential care model, revealing a significant increase in quality of life in the experimental group after six months of intervention, compared to the group receiving standard care provided in a residential setting. However, few studies comprehensively address a series of basic aspects of older adults’ well-being. Both loneliness and social isolation are complex to address and have profound implications for their physical, mental, and social well-being. Therefore, comprehensive approaches that combine individual, relational, and contextual factors are needed [11]. Therefore, our study aims to develop and evaluate a multicomponent intervention designed to address multiple domains simultaneously.

The accumulated evidence linking psychosocial variables to increased mortality risk underscores the need not only for detection strategies but also for targeted intervention programs. That is why including specific interventions beyond psychological support would be positive for users and would help prevent more serious health problems from appearing or worsening. In this sense, multi-domain or multicomponent interventions that combine different therapeutic approaches to improve health, well-being, or functional capacity have shown effectiveness in clinical, social, and community settings [19].

This modality integrates different types of interventions (physical, cognitive, emotional, social, nutritional, etc.), adapting to the individual needs of individuals and/or groups. They typically consider the individual from a comprehensive (biopsychosocial) perspective, and their effectiveness is usually greater than that of single interventions, especially in complex populations such as older adults or those with chronic illnesses. We find examples in the literature in the field of mild cognitive impairment (MCI) in older adults [20], where the authors, through a randomized controlled trial that included aerobic activity, strength training, balance, and dual tasks, found a significant improvement in cognitive and physical function. A recent Cochrane review conducted by Hafdy et al. [21] concluded that multi-domain interventions can be effective in preventing cognitive decline and dementia in older adults. Or the PsicAP program, a multicomponent intervention for the treatment of emotional disorders in primary care in Spain through transdiagnostic group cognitive-behavioral therapy [22, 23].

Despite growing interest in multimodal and technology-assisted interventions for older adults, evidence regarding their real-world effectiveness across multiple outcome domains remains limited. Previous studies have often focused on isolated components, single endpoints, or short-term effects, with less attention to longitudinal patterns of change in heterogeneous care settings. In this context, the present study adopts a pragmatic and exploratory approach to examine the longitudinal effects of a multimodal, technology-assisted intervention in older adults living both in the community and in residential care.

By assessing changes across cognitive, emotional, functional, and quality-of-life domains using linear mixed-effects models, this study evaluates the potential benefits of a multicomponent video call intervention over time. It also seeks to provide a more nuanced understanding of intervention-related changes under routine care conditions. We hypothesized that after applying a multimodal approach based the aforementioned system for a period of 18 months, the participants would show significant improvements in cognition, depression, loneliness, and QoL, but not in IADL or balance.

## Materials and methods

### Sample

Participants were recruited through publicly managed social service centers and private homes within the Community of Madrid (Comunidad de Madrid). Institutional settings included exclusively public facilities owned and operated by the Regional Government of Madrid, selected intentionally to enhance sample homogeneity and reduce socioeconomic bias. Admission to these centers follows standardized public dependency criteria, which supports representativeness and facilitates generalization of findings to the broader population of nursing home and day-center users. Community-dwelling participants were recruited from two socioeconomically and geographically contrasting areas (Northern and Southern zones of Madrid) to maximize external validity while maintaining feasibility constraints related to project resources and implementation logistics. The Northern zone is characterized by lower population density and predominantly single-family housing with higher income levels, whereas the Southern zone presents high-density urban environments, greater social vulnerability, and a higher prevalence of cognitive impairment. Recruitment and eligibility screening were coordinated through official Social Services and municipal networks to minimize self-selection bias. Reference social workers, familiar with participants’ clinical and social histories, conducted the initial identification and pre-screening of eligible candidates according to predefined inclusion criteria. Participants from nursing homes and community settings were analyzed jointly.

### Inclusion and exclusion criteria

The inclusion criteria were: be over 60 years of age or over 55 years of age with a diagnosed pathology and preferably with a recognized physical and/or cognitive dependency; have the cognitive capacity to maintain fluid conversations and repeat movements through a video call with a professional; be able to acquire the ability to connect autonomously to sessions or have a reference person in charge of the connections; the person must have a television with an HDMI input port. Exclusion criteria were: severe cognitive impairment, situations of great physical dependence that lack help, or psychotropic substance abuse or dependence.

### Ethics

The study was carried out in accordance with the Declaration of Helsinki. The protocol used in this study was approved by the Community of Madrid Research Ethics Committee on June 29, 2023 (protocol 3243/2023). Each participant was duly informed about the objectives of the study and expressed their agreement by signing the informed consent.

### Randomization

Simple randomization was used for participant allocation. Once the list of eligible subjects was compiled, each one was assigned a unique number. An independent assistant research generated a sequence of random numbers (1 for the control group and 2 for the experimental group). Then, one of the authors (S.G.-S.) assigned the participants to their respective groups ensuring allocation concealment. Randomization was not stratified by residential setting (home vs. nursing home) or by dependency level. Blinding of assessment was also guaranteed since the evaluation of results was performed by another researcher (G.P.-N.) without any knowledge of group assignment. Subject and clinician blinding was not possible due to the nature of intervention.

### Study design

**Fig. 1:**
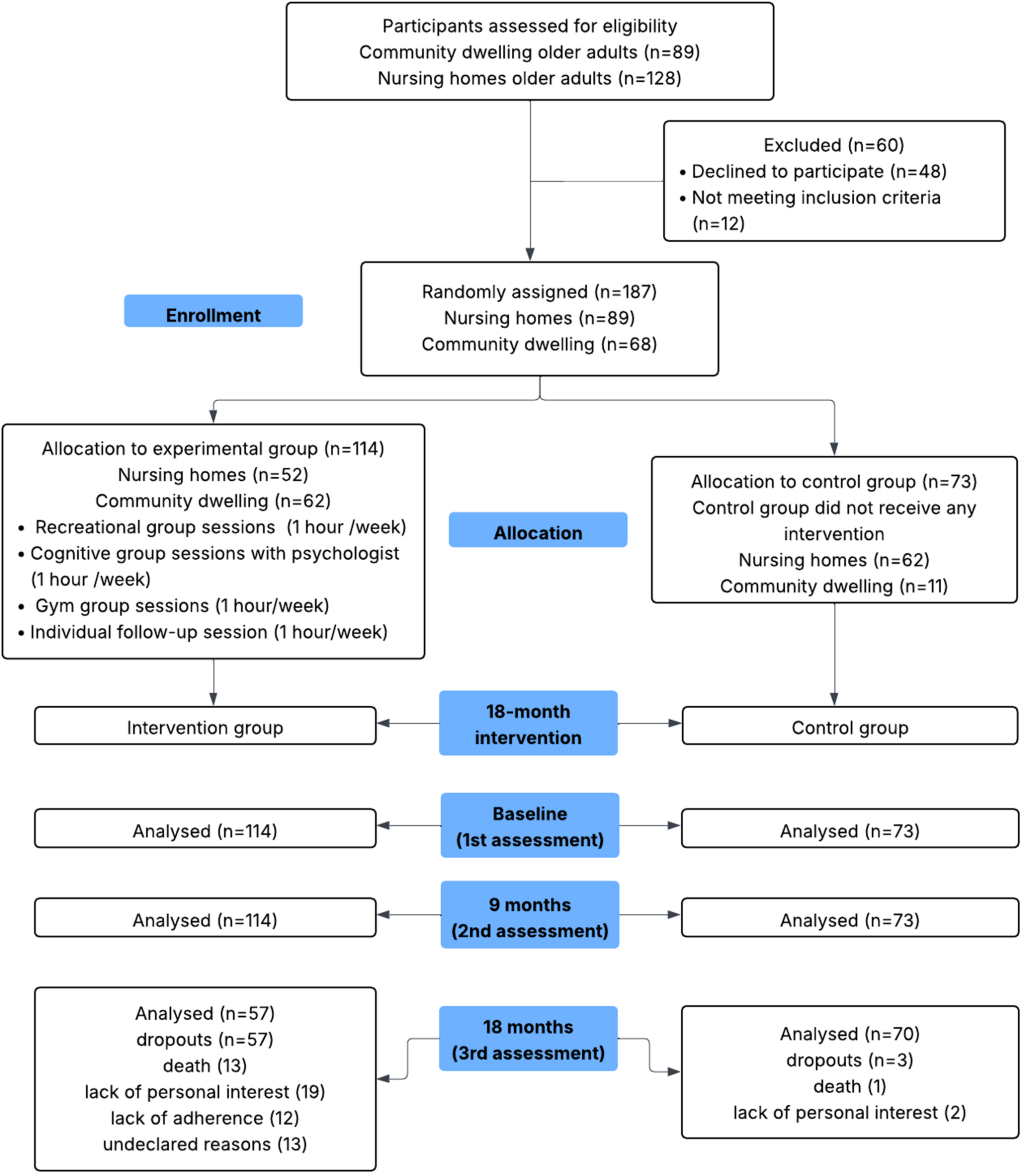
CONSORT diagram for the intervention

The study is a two-arm, parallel-group quantitative randomized control trial (RCT) with a longitudinal pre-test, mid-test and post-test within and between subject design. It was conducted between July 2023 and December 2024. This study was developed under a multicenter basis, since the intervention has involved institutionalized participants in two different nursing homes/care facilities as well as participants in community-dwelling settings. One hundred and eighty-seven participants were finally recruited. Attrition rates differed substantially between groups at the final 18-month follow-up. In the experimental group, 57 of 114 participants (50.0%) did not complete the final assessment, whereas 3 of 73 participants (4.1%) were lost in the control group. The main reasons for dropout in the experimental group were death (*n* = 13), lack of personal interest (*n* = 19), lack of adherence (*n* = 12), and undeclared reasons (*n* = 13). In the control group, reasons included death (*n* = 1) and lack of personal interest (*n* = 2). See Fig. 1 to check the full procedure of recruitment.

### Outcome measurements

The following inventories were used as instruments to support the feasibility and potential benefits of the intervention. To cognitively assess the participants, the MMSE [24] was used. The FUMAT scale [25] was used to assess quality of life by means of 8 different dimensions. The abbreviated version of the Yesavage Geriatric Depression Scale [26] was used to assess depression. Loneliness was assessed using the revised ESTE scale (ESTE-R) [27]. The Barthel’s scale [28] was used to measure the subjects’ functional capacity to perform basic activities and tasks of daily living (ADL) whereas the Lawton and Brody’s test [29] was used to measure the ability to perform instrumental activities of daily living (IADL). Finally, Tineti’s scale [30] was used to assess the balance and gait of older adults and determine the risk of falls.

### System description

**Fig. 2:**
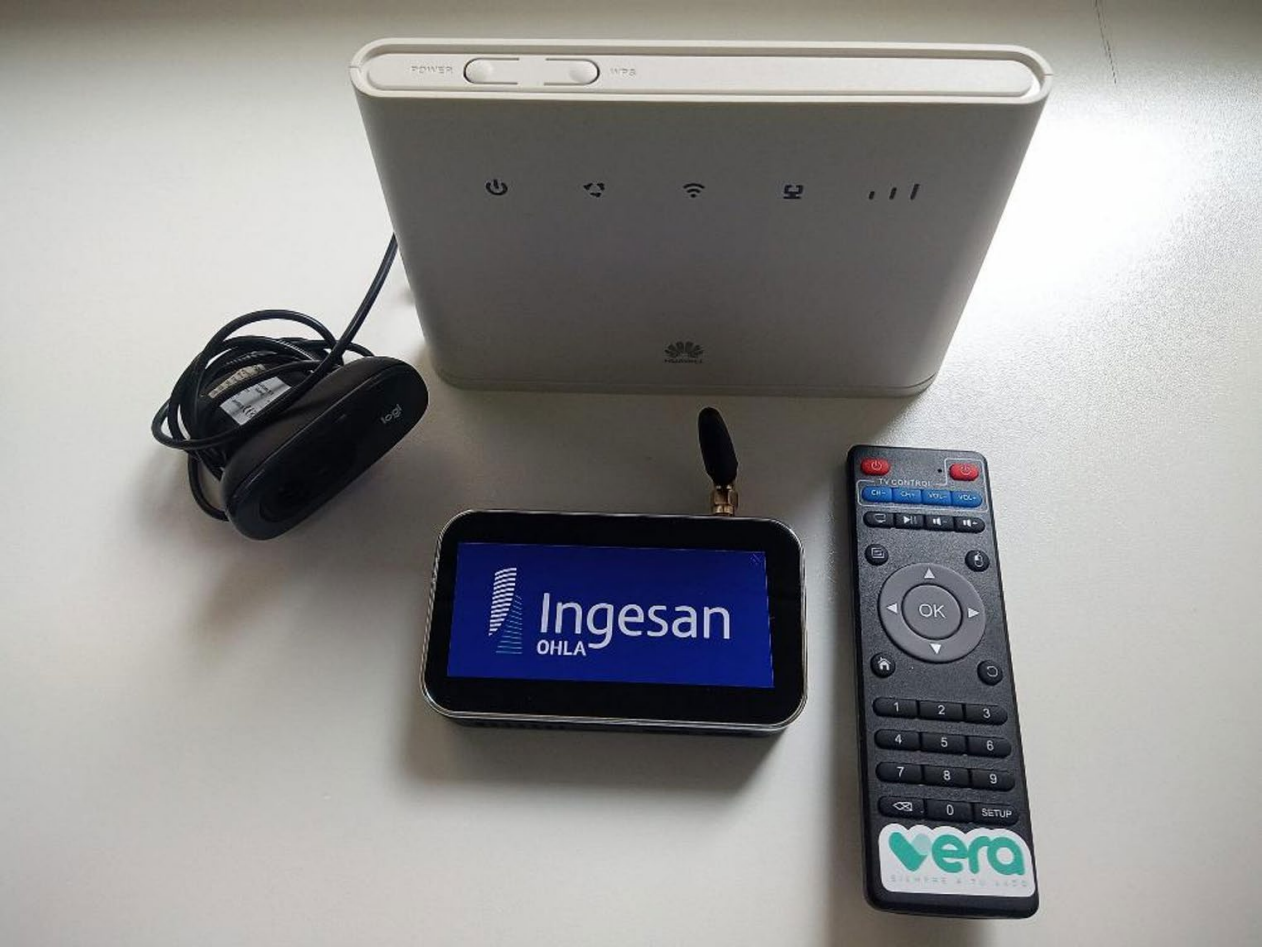
Required equipment for operating the Vera system

VERA is a virtual social center developed by OHLA-INGESAN, designed to replicate the functions of a traditional social center within users’ homes. It uses a simple plug-and-play setup connected via HDMI to the user’s TV, including a camera, remote control, and, if needed, a router (see Fig. 2). The technological system was supported by a team of professionals, including psychologists, social workers, physical therapists, and sociocultural coordinators, among others, who established care plans based on each individual’s specific needs. Once the video call system was installed, both the therapists conducting sessions in nursing homes and the subjects at home had permanent access to a technician to resolve any connectivity issues, who addressed them in real time. Users participated in individual and group sessions via a videoconference channel on their own television.

### Methodology

In the initial phase of the intervention, participants were evaluated using the different tests (baseline). The intervention was completely delivered via video call, by which the users received weekly sessions (individual and group). The control group simply continued their usual daily activities without any intervention.

Participants were classified according to the following therapeutic profiles: active aging, emotional disorders, unwanted loneliness, residential care center users, cognitive impairment, physical limitations and social exclusion. The assignment of therapeutic profiles was carried out following a comprehensive initial assessment process conducted by a multidisciplinary team comprised of professionals in social work, psychology, and physiotherapy. Best practices for assessing and diagnosing the personal circumstances of the participants, as required by the social and healthcare intervention protocols mandated by public administrations in Spain, were followed. The official dependency assessment for these individuals was also obtained.

Based on the information obtained, each participant was assigned to one or more non-mutually exclusive therapeutic profiles. The profiles were used as a tool for intervention planning, not as rigid categories. Belonging to a specific profile determines the therapeutic objectives, establishing the type, frequency, and intensity of assigned sessions best suited to addressing those needs, as outlined in each participant’s Individual Care Plan. Thus, profiles with greater emotional or social complexity received a higher proportion of individual and follow-up sessions, while profiles focused on active aging generally participated in preventive and recreational group sessions. The frequency of sessions was reviewed periodically and flexibly adjusted based on the user’s progress and level of participation.

The number of sessions assigned to each user was initially predetermined based on the therapeutic profile identified during the initial assessment. The intensity framework based on the starting profiles was as follows: active aging (3 weekly group sessions, 1 monthly follow-up session), unwanted loneliness and social exclusion (4 weekly group sessions, 1 weekly follow-up session), emotional disorders (2–3 weekly group sessions, 1 weekly follow-up session), cognitive impairment and physical limitation profiles (3 weekly group sessions, 1 monthly follow-up session). Intervention adherence was high across profiles. Among community-dwelling participants, 8,370 out of 9,984 planned sessions were delivered (84%), while in residential settings 3,251 out of 3,412 sessions were delivered (95%). Across the entire experimental sample, 11,621 of 13,396 planned sessions were completed, corresponding to an overall adherence rate of 87%. Session frequency and content were flexibly adapted to participants’ clinical and contextual needs according to predefined therapeutic profiles. Reasons for missing sessions included: death, physical, and emotional status, as well as to contextual factors such as fatigue, illness, or scheduling constraints. Table 1 shows the schedule of a typical weekly intervention for one of the participants, detailing the scheduled activities and their duration. The average weekly dose across participants was 3.75 h/week.

**Table 1:** Schedule corresponding to a typical weekly session within the intervention program for one participant. Individual sessions were held on a fortnightly basis

Time	Monday	Tuesday	Wednesday	Thursday	Friday
12:00–13:00	Recreational group session (60 min)		Cognitive group session with psychologist (60 min)		Individual follow-up session with social worker/ psychologist (60 min/monthly)
17:00–18:00				Gym group session with physiotherapist (60 min)	

Cognitive sessions included tasks to develop executive functions, logical thinking, and problem solving such us memory games, visual attention exercises, etc. For the recreational sessions, activities such as bingo, workshops about music, healthy eating, short stories, cooking, etc. were carried out in order to cover the different subscales of the FUMAT scale. Physical activities included joint mobility exercises, upper and lower limb muscle strengthening, balance and coordination exercises aimed at fall prevention, as well as breathing and body awareness exercises. The weekly follow-up sessions with the social worker focused primarily on providing ongoing psychosocial support, whereas the sessions with the psychologist centered on emotional support and psychological well-being.

In order to verify our hypothesis, participants were re-evaluated approximately halfway through the intervention and at the end of the intervention. Therefore, in addition to the baseline measures, two more measures were obtained.

### Statistical analysis

Data were reported as mean ± standard deviation (SD). Baseline demographic and clinical characteristics were compared between groups using chi-square tests for categorical variables and independent-samples t tests for continuous variables.

Longitudinal changes in outcome measures were analyzed using baseline-adjusted linear mixed-effects models with subject-specific random intercepts to account for within-individual correlation across repeated observations. Fixed effects included group (GROUP), time of assessment (TIME), and their interaction (GROUP × TIME). To further address baseline imbalances observed in some variables, the baseline value of each outcome was included as a covariate in the corresponding model. Given the unequal distribution of participants across residential contexts, residential setting (nursing home vs. community-dwelling) was included as an additional fixed-effect covariate in all models to account for potential contextual differences between settings. Model parameters were estimated using restricted maximum likelihood (REML). This modeling strategy allows robust estimation of longitudinal effects without requiring the assumption of sphericity and appropriately accounts for interindividual heterogeneity.

Model adequacy was documented using − 2 restricted log likelihood values and information criteria (AIC and BIC) provided by the SPSS MIXED procedure. Residual distributions were examined graphically to evaluate normality and homoscedasticity assumptions. Visual inspection indicated no substantial deviations from model assumptions.

Analyses followed an intention-to-treat principle, including all randomized participants with available data. Missing observations were handled within the baseline-adjusted linear mixed-effects modeling framework, which incorporates all available data under a missing-at-random assumption without the need for ad hoc imputation procedures. To evaluate potential attrition bias, baseline demographic and clinical characteristics were compared between participants who completed the 18-month follow-up and those who dropped out using independent-samples t tests.

Effect sizes were reported as fixed-effect estimates (β) derived from the baseline-adjusted linear mixed-effects models. Standardized effect sizes were calculated by dividing the group × time interaction coefficients by the residual standard deviation of each model, following a model-based standardization approach. All standardized effects were derived from the baseline-adjusted models. These values represent model-based standardized effects rather than conventional Cohen’s d calculated from pooled standard deviations. Standardized effects were interpreted using conventional thresholds: small (0.20–0.49), moderate (0.50–0.79), and large (≥ 0.80), with values below 0.20 considered negligible. Confidence intervals for fixed-effect estimates are reported in the Results section.

All statistical analyses were performed using SPSS version 26 (IBM Corp., Armonk, NY), and statistical significance was set at *p* < .05.

## Results

### Baseline data

The average age of the participants was 83.23 years (SD = 8.58), with ages ranging from 56 to 101 years. Regarding gender, 31.02% were men (58) and 68.98% women (129). At baseline, the groups were homogeneous in terms of gender, age, age > = 80 years. Demographic data (gender and age) and test scores at baseline are presented in Table 2.

**Table 2:** Demographics of recruited participants (*N* = 187)

	Total (*N* = 187)	Control group (*N* = 73)	Experimental group (*N* = 114)	*P* value
Age, years, mean (SD)	83.23 (8.58)	83.81 (8.93)	82.86 (8.37)	0.463
Age > = 80 years, N (%)	130 (69.51)	51(69.86)	79 (69.29)	0.089
Gender, Female, N(%)	129 (68.98)	45 (61.64)	84 (73.68)	0.736
MMSE	25.06 (7.72)	23.64 (6.96)	25.96 (8.07)	0.006
GDS	4.51 (3.00)	4.51 (2.73)	4.52 (3.17)	0.864
LAWTON	5.14 (2.11)	4.93 (1.72)	5.28 (2.32)	0.152
FUMAT	86.19 (12.89)	83.11 (11.92)	88.16 13.16)	0.003
ESTE-R	12.65 (6.19)	12.90 (5.18)	12.48 (6.77)	0.432
BARTHEL	73.75 (25.52)	66.99 (24.04)	78.08 (25.60)	< 0.001
TINETI	17.75 (7.15)	15.45 (6.99)	19.22 (6.88)	< 0.001

Values are presented as mean (standard deviation) or number (%). MMSE: Minimental State Examination; GDS: Yesavage Geriatric Depression Scale; Lawton: Lawton and Brody’s test; FUMAT: Fumat scale; ESTE-R: the revised ESTE scale; Barthel: Barthel’s scale; Tinetti: Tineti’s scale. Baseline p-values are provided for descriptive purposes only and were not adjusted for multiple comparisons

*p < .05, statistic significant

### Missing data and attrition

To examine the potential presence of attrition bias, baseline demographic and clinical characteristics were compared between completers and non-completers regardless of group allocation. Independent-samples t tests showed no statistically significant differences at baseline in age, MMSE, GDS, FUMAT, ESTE-R, Lawton Index, Barthel Index, or Tinetti scores (all *p* > .05) (Table 3). These findings suggest that dropout was not systematically associated with initial clinical status.

**Table 3:** Baseline Characteristics of Completers and Dropouts at 18-Month Follow-Up

Variable	Dropouts (*n* = 61) Mean ± SD	Completers (*n* = 126) Mean ± SD	t	*p*
Age (years)	81.72 ± 9.81	83.96 ± 7.87	−1.68	0.095
MMSE	25.17 ± 7.57	25.33 ± 7.17	−0.14	0.892
GDS	4.82 ± 2.89	4.73 ± 2.90	0.20	0.845
FUMAT	84.26 ± 14.02	87.15 ± 12.30	−1.44	0.152
ESTE-R	13.30 ± 6.94	12.37 ± 5.87	0.96	0.340
Lawton Index	5.08 ± 2.33	5.17 ± 2.01	−0.28	0.780
Barthel Index	72.31 ± 28.91	74.44 ± 23.81	−0.54	0.593
Tinetti	16.77 ± 7.39	18.22 ± 7.02	−1.30	0.194

Given the longitudinal design and the presence of incomplete repeated-measures data, all primary analyses were conducted using baseline-adjusted linear mixed-effects models estimated via restricted maximum likelihood. This modeling framework allows inclusion of all available observations without the need for single-value imputation and provides unbiased estimates under the missing-at-random assumption. Therefore, participants with incomplete follow-up data contributed all available data points to the analysis.

Taken together, these findings indicate that although attrition was higher in the experimental group at the final assessment, baseline comparability between completers and dropouts reduces concern regarding systematic attrition bias affecting the main longitudinal results.

The results derived from the baseline-adjusted linear mixed-effects models are presented below. Longitudinal changes in outcome measures were analyzed using linear mixed-effects models with subject-specific random intercepts to account for within-individual correlation across repeated observations. Fixed effects included group (GROUP), time of assessment (TIME), and their interaction (GROUP × TIME), while the baseline value of each outcome was included as a covariate to control for initial group differences. Model parameters were estimated using REML. Results are presented separately for each outcome variable.

### General cognitive state

The baseline-adjusted linear mixed-effects model revealed a significant group × time interaction for MMSE scores (F(2, 335.12) = 12.57, *p* < .001). Compared with the control group, the experimental group showed a significantly greater improvement of 1.58 points at mid-intervention (β = 1.58, SE = 0.32, 95% CI [0.96, 2.21], *p* < .001) and 1.07 points post-intervention (β = 1.07, SE = 0.32, 95% CI [0.45, 1.70], *p* = .001). The random-effects structure indicated inter-individual variability, with a significant subject-level random intercept variance (0.58, *p* < .001), while the residual variance was relatively small (1.78, *p* < .001). These effects correspond to large standardized effect sizes (d ≈ 1.19 at mid-intervention and d ≈ 0.80 at post-intervention), indicating a clinically meaningful improvement in cognitive performance.

### Depression

For depressive symptoms measured by the GDS, the baseline-adjusted linear mixed-effects model revealed a significant main effect of time on GDS scores (F(2, 315.55) = 7.09, *p* = .001), indicating overall changes in depressive symptoms across assessment points. In addition, a significant group × time interaction was observed (F(2, 315.72) = 16.41, *p* < .001), reflecting differential longitudinal trajectories between the experimental and control groups. Compared with the control group, the experimental group showed a significantly greater reduction of 1.32 points in depressive symptoms at mid-intervention (β = −1.32, SE = 0.25, 95% CI [− 1.81, − 0.82], *p* < .001), whereas the group difference at post-intervention was not statistically significant (β = −0.34, SE = 0.25, 95% CI [− 0.84, 0.16], *p* = .177). The effect observed at mid-intervention corresponded to a large standardized effect size (d ≈ 1.29), reflecting a clinically meaningful reduction in depressive symptoms in the experimental group. At the second follow-up, the difference between groups was small and not statistically significant (d ≈ − 0.34). Overall, these results suggest that the intervention was associated with a pronounced early reduction in depressive symptoms, followed by a partial convergence of group scores over time.

### Quality of life

For quality of life measured by the FUMAT scale, the baseline-adjusted linear mixed-effects model revealed a significant group × time interaction (F(2, 326.58) = 75.41, *p* < .001), indicating differential longitudinal changes in quality of life between the experimental and control groups after adjusting for baseline scores. Compared with the control group, the experimental group showed a significantly greater improvement of 5.62 points in FUMAT scores at mid-intervention (β = 5.62, SE = 0.47, 95% CI [4.69, 6.55], *p* < .001) and 2.43 points at post-intervention (β = 2.43, SE = 0.47, 95% CI [1.50, 3.35], *p* < .001). These effects corresponded to extremely large standardized effect sizes at mid-intervention (d ≈ 5.51) and very large effect sizes at post-intervention (d ≈ 2.38), indicating a substantial and clinically meaningful improvement in quality of life in the experimental group, particularly during the early phase of the intervention.

### Perceived loneliness

For loneliness measured by the ESTE scale, the baseline-adjusted linear mixed-effects model revealed a significant group × time interaction (F(2, 342.83) = 31.09, *p* < .001), indicating differential longitudinal changes between the experimental and control groups. Compared with the control group, the experimental group showed a significantly greater reduction of 2.88 points in loneliness at mid-intervention (β = −2.88, SE = 0.37, 95% CI [− 3.61, − 2.14], *p* < .001) and 1.25 points at post-intervention (β = −1.25, SE = 0.37, 95% CI [− 1.99, − 0.52], *p* = .001). These effects corresponded to very large standardized effect sizes at mid-intervention (d ≈ 2.16) and large effect sizes at post-intervention (d ≈ 0.94), indicating a substantial reduction in perceived loneliness in the experimental group, particularly during the early phase of the intervention.

### Functional capacity (ADL and iADL)

The baseline-adjusted linear mixed-effects model showed no significant main effects of group on Lawton Index scores (F(1, 203.43) = 0.09, *p* = .762) or time (F(2, 334.11) = 1.72, *p* = .181), nor a significant group × time interaction (F(2, 334.17) = 0.19, *p* = .824), indicating stable instrumental functional independence across assessment points in both groups. In line with these findings, none of the group × time interaction contrasts reached statistical significance at either mid-intervention (β = 0.04, SE = 0.09, 95% CI [− 0.14, 0.22], *p* = .684), corresponding to a between-group difference of 0.04 points, or post-intervention (β = 0.06, SE = 0.09, 95% CI [− 0.12, 0.24], *p* = .535), corresponding to 0.06 points. Consistently, the associated effect sizes were negligible at both mid-intervention (d ≈ 0.03) and post-intervention (d ≈ 0.04), indicating no clinically meaningful differential effect of the intervention on instrumental activities of daily living.

On the other hand, for basic activities of daily living measured by the Barthel Index, the baseline-adjusted linear mixed-effects model revealed a significant group × time interaction (F(2, 333.94) = 4.11, *p* = .017), indicating differential longitudinal changes between the experimental and control groups. Compared with the control group, the experimental group showed a significantly greater improvement of 2.95 points at mid-intervention (β = 2.95, SE = 1.04, 95% CI [0.91, 5.00], *p* = .005), whereas the group difference at post-intervention was not statistically significant (β = 1.53, SE = 1.04, 95% CI [− 0.52, 3.57], *p* = .143), corresponding to 1.53 points. These effects corresponded to a very large standardized effect size at mid-intervention (d ≈ 2.22) and a large but non-significant effect at post-intervention (d ≈ 1.15), suggesting an early functional improvement with partial convergence over time.

### Physical condition and autonomy

The baseline-adjusted linear mixed-effects model did not reveal a significant main effect of group on Tinetti scores (F(1, 203.46) = 1.92, *p* = .168). Similarly, neither the main effect of time (F(2, 333.16) = 1.62, *p* = .200) nor the group × time interaction (F(2, 333.55) = 0.58, *p* = .561) reached statistical significance, suggesting no differential longitudinal change between groups. Consistent with these findings, the group × time interaction estimates were not statistically significant at either mid-intervention (β = 0.25, SE = 0.25, 95% CI [− 0.23, 0.74], *p* = .302), corresponding to 0.25 points, or post-intervention (β = 0.10, SE = 0.25, 95% CI [− 0.38, 0.58], *p* = .687), corresponding to 0.10 points. The resulting effect sizes were small at both mid-intervention (d ≈ 0.19) and post-intervention (d ≈ 0.08), indicating no meaningful intervention-related effect on balance and gait performance as measured by the Tinetti scale.

To summarize the intervention effect, Table 4 shows the standardized effect sizes derived from baseline-adjusted linear mixed-effects models. Positive values indicate greater improvement in the experimental group, whereas negative values indicate a greater reduction in symptom severity (e.g., GDS and ESTE-R scores). Standardized effect sizes (d) were calculated by dividing the group × time interaction coefficients (β) by the residual standard deviation of each model. Mid-intervention and Post-intervention correspond to the first and second follow-up assessments relative to baseline.

**Table 4:** Model-Based Standardized Effect Sizes Derived from Baseline-Adjusted Linear Mixed-Effects Models

Outcome	Group × Mid-intervention (β)	d (Mid-intervention)	Magnitude	Group × Post-intervention (β)	d (Post-intervention)	Magnitude
MMSE	1.58	1.18	Large	1.07	0.80	Large
GDS	−1.32	1.27	Large	−0.34	0.33	Small
FUMAT	5.62	5.51	Very large	2.43	2.38	Very large
ESTE-R	−2.88	2.16	Very large	−1.25	0.94	Large
BARTHEL	2.95	2.22	Very large	1.53	1.15	Large (ns)
LAWTON	0.04	0.03	Negligible	0.06	0.04	Negligible
TINETTI	0.25	0.19	Small	0.10	0.08	Negligible

Standardized effect sizes (d) were calculated by dividing the group × time interaction coefficients (β) by the residual standard deviation of the corresponding baseline-adjusted linear mixed-effects model. These values represent model-based standardized effects rather than conventional Cohen’s *d* calculated from pooled baseline standard deviations. 95% confidence intervals are reported for the fixed-effect estimates (β) in the Results section, from which the standardized effects were derived

### Subgroup analysis by residential setting

Given the unequal distribution of participants across residential contexts, all models were adjusted for residential setting (nursing home vs. community-dwelling). The inclusion of residential setting as a covariate did not materially alter the pattern or magnitude of the group × time effects across outcomes. Intervention-related improvements in cognition (MMSE), depressive symptoms (GDS), perceived loneliness (ESTE), quality of life (FUMAT), and basic activities of daily living (Barthel) remained statistically significant after adjustment. No significant intervention effects were observed for instrumental activities of daily living (Lawton) or balance and gait performance (Tinetti), consistent with the primary analyses.

Residential setting was not significantly associated with most outcomes and did not modify the intervention effect. A significant main effect of residential setting was observed only for Barthel scores, with nursing home participants demonstrating lower overall functional levels; however, this did not influence the longitudinal intervention-related changes.

## Discussion

This study examined whether a multimodal intervention delivered via a video call system over an 18-month period could improve a range of clinically validated outcomes in older adults receiving home-based or residential care. Building on a previously published pilot study conducted in a very small sample [19], the present work extends those preliminary findings using a sufficiently large sample to allow robust longitudinal analysis through baseline-adjusted linear mixed-effects models.

Overall, the results indicate a domain-specific pattern of effectiveness rather than a uniform intervention effect. Favorable longitudinal changes were observed in cognitive functioning, depressive symptoms, perceived loneliness, quality of life, and basic functional capacity. Effect sizes ranged from small to substantial within the mixed-model framework across outcome domains and time points. Particularly strong effects were observed in quality of life (FUMAT) and perceived loneliness (ESTE-R), which showed large model-based standardized effects at mid-intervention and sustained large effects at post-intervention. Cognitive performance (MMSE) and depressive symptoms (GDS) demonstrated large effects at mid-intervention, with partial attenuation over time but maintained clinical relevance. Basic functional capacity (Barthel Index) also showed very large effects at mid-intervention and large effects at follow-up. In contrast, instrumental activities of daily living (Lawton Index) and balance and gait performance (Tinetti) showed negligible or small effects, indicating limited responsiveness to the intervention in these domains.

Importantly, these effects remained significant after adjustment for baseline values in the mixed-effects models, indicating that the observed improvements were not attributable to initial group differences or contextual factors. No evidence suggested differential intervention responses between nursing home and community-dwelling participants, and the overall pattern of effects was comparable across settings. Although expected differences in overall functional levels were observed between residential contexts, these did not influence the longitudinal intervention-related changes.

Given the inclusion of multiple outcome domains, the present study should be interpreted as exploratory in nature. No formal correction for multiplicity was applied, in line with the exploratory and multidimensional objectives of the trial. Therefore, the findings should be interpreted in terms of overall consistency and coherence across related domains. They should not be reduced to isolated statistical significance tests. These results suggest that sustained, technology-mediated multimodal interventions may produce clinically meaningful benefits in cognitive, emotional, and psychosocial domains in older adults.

It is important to acknowledge that attrition rates were higher in the experimental group at the final follow-up. However, baseline comparisons between completers and dropouts did not reveal significant differences in demographic or clinical variables, suggesting that dropout was not systematically associated with initial health status or outcome severity. While differential attrition between groups may introduce some uncertainty in long-term estimates, the use of linear mixed-effects models allowed inclusion of all available data under a missing-at-random assumption, thereby minimizing potential bias associated with incomplete follow-up. Nevertheless, the imbalance in attrition rates should be considered when interpreting long-term intervention effects, particularly at the final assessment point.

In contrast, no meaningful intervention-related changes were detected in instrumental activities of daily living or balance and gait performance. These null findings are consistent with the characteristics of the intervention, which included a remote and relatively low-intensity physical component that was not specifically designed to provide intensive, task-specific, or supervised functional training.

To sum up, and from a clinical perspective, the magnitude of the observed changes suggests meaningful functional and psychosocial benefits. The approximately 1.5-point improvement in MMSE at mid-intervention represents a modest but potentially relevant cognitive gain in older adults at risk of decline, where even small improvements may reflect stabilization or delayed deterioration over time [31]. Similarly, the reduction of about 1.3 points in GDS scores indicates a noticeable decrease in depressive symptom burden, consistent with evidence that modest reductions in late-life depressive symptoms may translate into meaningful improvements in daily functioning and engagement [32]. The nearly 3-point reduction in perceived loneliness (ESTE) and the 5–6-point improvement in quality of life (FUMAT) reflect substantial psychosocial benefits aligned with prior evidence linking reductions in perceived social isolation to improved well-being and health outcomes [33, 34]. In functional terms, the approximately 2.8-point increase in Barthel Index scores suggests improved independence in basic activities of daily living, with prior work indicating that small changes in Barthel scores may correspond to clinically meaningful functional shifts in older or clinically vulnerable populations [35]. However, the absence of effects in instrumental activities (Lawton) and balance and gait performance (Tinetti) indicates that higher-level autonomy and mobility outcomes may require more intensive or targeted physical interventions.

Taken together, the results highlight the importance of considering outcome-specific responsiveness when evaluating multimodal interventions. While remote, technology-based programs appear well suited to improving cognitive, emotional, and quality-of-life outcomes, more targeted and intensive strategies may be required to achieve measurable gains in complex functional domains. These findings have relevant implications for the design of future hybrid or stepped-care interventions aimed at promoting healthy aging and autonomy in older adults.

### General cognitive state

The baseline-adjusted linear mixed-effects models revealed a sustained and clinically meaningful intervention effect on global cognitive functioning. Cognitive performance, as measured by the MMSE, showed large standardized effects at both mid-intervention and post-intervention, reflecting a differential longitudinal trajectory between groups. While the control group exhibited progressive cognitive decline across follow-up assessments, the experimental group demonstrated relative stability and improvement, indicating that participation in the multimodal intervention was associated with preservation of cognitive functioning over time.

Importantly, the observed cognitive changes may not be attributable solely to direct cognitive stimulation. Improvements in mood and reductions in perceived loneliness, both of which showed strong effects in the present study, together with increased opportunities for social interaction, may have indirectly contributed to cognitive outcomes. Previous research has highlighted the close relationship between depressive symptoms, social isolation, and cognitive functioning in older adults [36–38]. In this context, the multimodal nature of the intervention—combining cognitive activities with social engagement and emotional support—may help explain the sustained pattern of cognitive benefit observed.

The link between physical exercise and cognitive stimulation may also play a relevant role. Tarazona-Santabalbina et al. [17] reported significant cognitive improvements in an intervention group alongside decline in controls following a multicomponent exercise program, a pattern consistent with the differential group × time trajectory observed in the present study. These findings align with evidence suggesting reciprocal relationships between motor activity and cognitive functioning [39]. Likewise, Song and Yu [40] and Langlois et al. [41] documented positive associations between physical activity and cognitive performance in older adults, supporting the notion that integrated, multimodal approaches may contribute to more favorable cognitive trajectories.

The magnitude and maintenance of the cognitive effect observed here are also consistent with studies emphasizing the importance of sufficient intervention duration and intensity. Tan et al. [16] reported that only participants exposed to a combined exercise and cognitive stimulation program showed significant cognitive improvements, as assessed by the MoCA, underscoring the importance of integrating multiple intervention components to achieve meaningful cognitive gains. Similarly, Lee et al. [42] observed significant improvements in MMSE scores following a long-term home-visiting cognitive intervention for older adults living alone. In line with the sustained large effects observed in the present study, participants with baseline cognitive impairment exhibited the greatest improvements. This suggests that individuals with greater vulnerability may benefit most from structured cognitive engagement.

In contrast, studies employing shorter intervention durations have reported more limited or inconsistent cognitive effects. Sok et al. [43] found significant cognitive changes only at the group × time interaction level following an 8-week multicomponent intervention, a duration that may be insufficient to induce robust and sustained cognitive change. Likewise, Chiang et al. [44] and Chen et al. [45] did not observe significant MMSE improvements after 12-week interventions, suggesting that short-term or low-intensity programs may not provide adequate exposure to produce measurable cognitive benefits. Collectively, these findings emphasize the importance of sufficient intervention duration and intensity when targeting cognitive outcomes.

The cognitive stimulation embedded within the present intervention may have played a key role in preserving cognitive performance over time. This is reflected in the large and sustained effects observed across follow-up assessments in the baseline-adjusted mixed-effects models. Previous research has shown that engaging in cognitively stimulating activities can help maintain cognitive function [46] and may reduce the risk of developing dementia [47], particularly in vulnerable populations [48]. Moreover, multimodal interventions that combine cognitive, physical, and social components have been shown to produce greater cognitive benefits than single-component approaches [49].

Importantly, the absence of cognitive engagement has been associated with accelerated cognitive decline in both cognitively healthy older adults and those with dementia (Salthouse, 2006). In this context, the present findings support the value of sustained, multimodal cognitive stimulation as a potential strategy to slow cognitive decline and promote cognitive resilience in older adults.

### Depression

Regarding depressive symptoms measured using the Yesavage Geriatric Depression Scale (GDS), the results indicate a significant reduction in depression primarily in the experimental group, characterized by a pronounced early improvement during the first phase of the intervention. The significant group × time interaction observed in the baseline-adjusted mixed-effects model suggests that changes in depressive symptoms followed different trajectories in the experimental and control groups. The most substantial between-group differences emerged at mid-intervention. Consistent with the model-based standardized estimates, a large effect was observed at mid-intervention, reflecting a clinically meaningful early reduction in depressive symptoms associated with participation in the intervention. In contrast, the control group did not exhibit significant changes in depressive symptoms over time.

Importantly, the pattern observed in this study suggests that depressive symptoms may be particularly responsive during the initial stages of a multimodal intervention.

The strong early reduction in GDS scores in the experimental group was followed by stabilization and partial convergence at later assessments. This pattern suggests that the intervention may exert its greatest emotional impact during the early phases, after which improvements are maintained rather than further amplified. This interpretation is supported by the attenuation of the standardized effect size at post-intervention, which decreased from large to small magnitude while remaining directionally favorable. This temporal pattern aligns with the notion that early engagement, novelty, and increased social contact may play a critical role in alleviating depressive symptoms in older adults [19].

These findings are consistent with previous studies reporting early and clinically meaningful reductions in depressive symptoms following multicomponent or socially oriented interventions. For example, Wang et al. [50] observed significant improvements in GDS scores with large effect sizes after a 12-week magic-based intervention among community-dwelling older adults.

Similarly, Tarazona-Santabalbina et al. [17] reported significant reductions in depressive symptoms relative to a control group following a multicomponent intervention. Tan et al. [16] also found significant improvements in depression within the first six months of a combined physical exercise and cognitive stimulation program. Comparable results were also reported by Lee and Lee [42], who documented significant reductions in depressive symptoms following a home-visiting cognitive intervention among older adults living alone. In line with these studies, the present results suggest that the emotional benefits of multimodal programs may emerge rapidly, particularly when social and interactive components are emphasized.

The observed reduction in depressive symptoms may be partially explained by the social and emotional components of the intervention. Online interaction and regular interpersonal contact have been proposed as important sources of emotional support, potentially mitigating feelings of loneliness and social isolation [51, 52]. As highlighted by Domènech-Abella et al. [53], both the size and quality of social networks play a crucial role in the relationship between loneliness and depression in older populations. In this context, the intervention implemented in the present study may have expanded participants’ social networks and increased opportunities for meaningful interaction, thereby contributing to the early reduction in depressive symptoms.

At the same time, not all short-term interventions have demonstrated comparable effects on depressive symptoms. For instance, Chen et al. [45] and Chiang et al. [44] did not observe significant changes in GDS scores following relatively brief cognitive stimulation interventions. These discrepancies suggest that intervention intensity, content, and the degree of social engagement may be critical factors influencing emotional outcomes. In particular, very limited intervention durations or low weekly exposure may be insufficient to produce measurable changes in depressive symptoms, especially in populations with more complex clinical profiles.

The temporal pattern observed in depressive symptoms highlights the importance of early responsiveness in emotional outcomes. The marked initial reduction suggests that older adults may be particularly sensitive to enhanced social contact and structured engagement during the first stages of intervention, whereas longer-term maintenance may depend on sustained motivational and contextual factors. Future studies with longer follow-up periods and more frequent early assessments would be valuable to further clarify the timing, durability, and mechanisms underlying these early improvements in mood.

### Perceived loneliness

The results of the present study indicate significant and clinically meaningful improvements in perceived loneliness, as measured by the ESTE-R scale, associated with participation in the multimodal intervention. The baseline-adjusted linear mixed-effects model revealed significant main effects of group and time, as well as a strong group × time interaction. This indicates differential longitudinal trajectories between the experimental and control groups. These findings suggest that changes in perceived loneliness were not only time-dependent but also strongly influenced by group membership.

Specifically, participants in the experimental group showed a pronounced reduction in perceived loneliness at mid-intervention, corresponding to a large model-based standardized effect within the mixed-effects framework. This was followed by a sustained—though attenuated—improvement at the post-intervention assessment, where the effect size remained in the large range. In contrast, no significant changes were observed in the control group over the course of the study. This temporal pattern indicates a strong early impact of the intervention on social-emotional loneliness, with gains that were largely maintained over time rather than continuing to increase. The magnitude of these effects places loneliness among the outcome domains most strongly influenced by the intervention.

These findings are consistent with previous studies that have implemented comprehensive intervention strategies designed by multidisciplinary teams. For example, Rodríguez-Romero et al. [15] reported a significant reduction in perceived loneliness among community-dwelling older adults following a multicomponent intervention, alongside improvements in depressive symptoms, social support, and the mental health component of quality of life.

Similarly, a recent review conducted by Yu et al. [54] highlighted the effectiveness of non-pharmacological interventions in reducing loneliness in older adults. While psychological interventions appear particularly effective, strategies that enhance social dynamics and interpersonal connectivity may also produce meaningful benefits. In line with these findings, the large model-based effects observed in the present study reinforce the potential of socially enriched multimodal programs to substantially reduce perceived loneliness in vulnerable populations.

However, not all multicomponent interventions have demonstrated comparable effects on loneliness. Hernández-Ascanio et al. [55], for instance, implemented an intervention aimed at stimulating social integration and renewed socialization but did not observe significant improvements in perceived loneliness. The authors attributed these findings to the intervention’s primary focus on social isolation rather than loneliness itself. As discussed in previous literature, loneliness and social isolation are related but distinct multidimensional constructs, each requiring different intervention approaches [56, 57]. Whereas interventions targeting social isolation may prioritize increasing opportunities for interaction, interventions addressing loneliness often require additional psychological and emotional components [58].

These considerations highlight the inherent difficulty of designing interventions capable of simultaneously addressing multiple psychosocial outcomes. In this context, the results of the present study suggest that a multimodal approach combining social participation, leisure engagement, and emotional support may be particularly suitable for reducing perceived loneliness. Evidence from a systematic review conducted by Smallfield and Molitor [59] supports the notion that active engagement in meaningful social and leisure activities can mitigate feelings of loneliness in older adults.

These findings support the interpretation that multimodal, technology-assisted interventions may be particularly effective in reducing perceived loneliness in older adults, especially during the early stages of implementation. This is evidenced by the substantial early model-based effect and the maintained large effect at final follow-up identified in the longitudinal mixed-model analysis. These benefits appeared to be sustained over time. Future research should further explore the specific mechanisms and intervention components responsible for these effects, as well as their long-term sustainability.

### Quality of life

Regarding quality of life, the FUMAT scale indicated statistically significant effects of group, time, and their interaction. Large to substantial model-based standardized effects were observed at both mid- and post-intervention assessments.

The baseline-adjusted linear mixed-effects model revealed a pronounced early improvement in quality of life in the experimental group, particularly between baseline and the first follow-up assessment. This was followed by a robust and sustained benefit at post-intervention, with effect sizes remaining in the upper large range within the mixed-model framework. In contrast, the control group showed no significant improvements over time and, in some dimensions, exhibited a deterioration in quality of life. This temporal pattern is consistent with findings reported by Palacios-Navarro et al. [19], who also observed the greatest gains during the initial phase of a multicomponent intervention.

Analysis of the FUMAT subscales showed that all dimensions of quality of life differed significantly between groups, with medium to large improvements observed in the experimental group. The magnitude and consistency of the global FUMAT effects suggest that quality of life was one of the domains most strongly influenced by the intervention. This supports the suitability of the FUMAT scale for capturing multidimensional changes in quality of life, as it provides a comprehensive profile that extends beyond health-related aspects alone. Unlike instruments such as the HRQOL-14 [60] or the EQ-5D [61], which primarily focus on health status and treatment effects, the FUMAT scale encompasses broader psychosocial and functional domains. This makes it particularly appropriate for evaluating complex, person-centered interventions.

The present findings are consistent with previous studies reporting improvements in quality of life following multicomponent or person-centered care interventions. Díaz-Veiga et al. [18] observed increased quality of life, measured with the FUMAT scale, in older adults with cognitive impairment receiving a person-centered care model. Similarly, Kousha et al. [51] reported significant improvements in quality of life following an intervention aimed at reducing loneliness among community-dwelling older adults, highlighting the role of social interaction and engagement. Other studies using different quality-of-life measures, such as Tarazona-Santabalbina et al. [17] and Song and Yu [40], have also documented greater improvements in experimental groups following multicomponent or physical exercise interventions. The very large effects observed in the present study reinforce the potential impact of sustained, multidomain programs when multiple psychosocial and functional components are addressed simultaneously.

However, not all interventions have demonstrated significant effects on quality of life. Studies with shorter duration or lower intensity, such as those conducted by Wang et al. [50] and Chiang et al. [44], did not observe significant improvements, suggesting that intervention intensity, duration, and multidimensional content may be critical factors influencing quality-of-life outcomes.

To sum up, the strength and consistency of the FUMAT findings suggest that quality of life may be particularly sensitive to sustained, multidomain interventions that simultaneously address cognitive, emotional, and social needs. Rather than reflecting isolated improvements in single domains, the substantial longitudinal effects observed here likely capture the cumulative impact of meaningful engagement, structured activity, and interpersonal connection. These results reinforce the importance of comprehensive, person-centered programs when the primary goal is to enhance global well-being in older adults.

### Functional capacity (ADL and iADL)

Regarding functional capacity, the results differed between basic and instrumental activities of daily living. For basic activities of daily living, assessed using the Barthel Index, the findings indicate significant improvements in the experimental group during the first phase of the intervention, particularly within the first nine months. The baseline-adjusted linear mixed-effects model revealed a very large standardized effect at mid-intervention. In contrast, the control group showed a less favorable trajectory, with a decline in functional scores observed toward the end of the follow-up period. This pattern is consistent with the significant group × time interaction identified in the mixed-effects model and suggests that the intervention was associated with early gains in basic functional independence. Although the effect size decreased at post-intervention, it remained in the large range but did not reach statistical significance. This suggests partial maintenance with some attenuation over time.

These results are in line with previous multicomponent intervention studies. Chen et al. [45], for example, reported significant improvements in Barthel scores following a multicomponent exercise program, while no significant changes were observed in instrumental activities of daily living measured by the Lawton and Brody Index. Although their intervention was relatively short (12 weeks), it placed strong emphasis on physical training, including aerobic, resistance, balance, and flexibility exercises, which may explain the observed improvements in basic functional capacity. Similarly, Tarazona-Santabalbina et al. [17] found significant improvements in both Barthel and Lawton and Brody scores following a high-intensity multicomponent exercise intervention, suggesting that intervention intensity and duration may play a key role in achieving broader functional gains. In the present study, the substantial early Barthel effect suggests that even remotely delivered, moderate-intensity programs may produce meaningful improvements in basic autonomy, particularly when sustained over time.

In contrast, no significant changes were observed in instrumental activities of daily living in the present study, as measured by the Lawton and Brody Index. The model-based standardized effects for iADL were negligible at both follow-up assessments, indicating an absence of meaningful differential change between groups. This finding is consistent with the results reported by Chiang et al. [44], who did not observe significant improvements in either ADL or iADL following a 12-week cognitive stimulation therapy. The authors attributed this lack of change partly to limitations in the ecological validity of functional scales within institutional settings, where participants may be unable to perform or accurately report certain instrumental activities.

More broadly, the literature suggests that improvements in instrumental activities of daily living are more difficult to achieve than gains in basic functional capacity. Several multicomponent interventions have failed to demonstrate statistically significant changes in iADL performance despite reporting improvements in physical capacity and cognitive functioning [62–64]. Instrumental activities often depend on higher-level cognitive processes, environmental opportunities, and social context, which may limit the transfer of gains from intervention settings to everyday life. This dissociation between basic and instrumental functional outcomes reinforces the need to tailor intervention components specifically toward ecologically grounded daily tasks if broader autonomy is the intended objective.

On the whole, these findings suggest that multimodal interventions may be particularly effective in supporting basic functional independence, while changes in instrumental activities of daily living may require longer intervention periods, higher intensity, or more ecologically targeted strategies. The dissociation observed between Barthel and Lawton outcomes highlights the importance of evaluating functional domains separately when assessing the impact of complex interventions in older adults.

### Physical condition and autonomy

The lack of improvement observed in Tinetti scores deserves specific consideration. The baseline-adjusted mixed-effects model showed only a small standardized effect at mid-intervention and a negligible effect at post-intervention, with no statistically significant group × time interaction. This indicates that balance and gait performance did not meaningfully change as a result of the intervention. Unlike cognitive, emotional, or quality-of-life outcomes, balance and gait performance typically require high-frequency, task-specific, and supervised physical training to elicit measurable changes. In the present study, the physical training component was delivered remotely and at a relatively low frequency, and was not primarily designed to directly target balance or gait through progressive, hands-on exercises.

Previous studies reporting significant improvements in Tinetti or related balance measures have generally employed in-person interventions with higher training frequency, direct supervision, and individualized progression, often including specific balance and gait tasks. For example, Espejo-Antúnez et al. [65] found significant improvements compared to the control group in areas such as functional mobility, musculoskeletal endurance, balance, gait, and risk of falls in institutionalized older adults. In that case, participants in the experimental group reached a total of 200 min per week (for 12 weeks) of physical exercise in their own residence, coordinated with a physical therapist. The same occurred in the study conducted by Tarazona-Santabalbina et al. [17], who found significant differences between the control and experimental groups in an intensive multicomponent intervention developed over a period of 24 weeks and five days per week. On the other hand, the controlled trial conducted by Castellote-Caballero et al. [66] included two sessions (each lasting 45 to 50 min) devoted to the physical component with direct supervision. The program was adapted to individual abilities to monitor progress more effectively. They found significant differences over time and in the group × time interaction. The group that received the combined physical–cognitive treatment experienced statistically significant differences between the pre- and post-measurement.

In contrast, remote or low-intensity physical activity programs have shown more limited effects on balance-related outcomes, even when other domains improve. For instance, Chen et al. [45] found no significant differences in the Tinetti score in the experimental group in their 12-week intervention, with only the control group performing significantly worse over time. The present findings therefore appear consistent with the broader literature suggesting that mobility-related outcomes are particularly sensitive to training intensity, supervision, and ecological specificity.

Future interventions aiming to improve balance and gait should consider incorporating higher-frequency, supervised, and task-specific physical training components, potentially delivered in person or through hybrid models, to maximize their impact on mobility-related outcomes. These results reinforce the importance of aligning intervention design with outcome-specific physiological demands when targeting complex motor functions in older adults.

From an implementation perspective, the translation of this type of multimodal, technology-mediated intervention into routine care settings requires careful consideration of practical constraints. Although the weekly time commitment was relatively modest, successful rollout would depend on adequate staff training and structured supervision protocols. Ongoing technical support would also be necessary to ensure participant engagement and system reliability. Initial investment costs related to digital infrastructure, device provision, and training may represent barriers, particularly in under-resourced care settings. Moreover, variability in digital literacy among older adults and care personnel may necessitate tailored onboarding strategies. Ensuring data security, privacy compliance, and long-term sustainability of technological platforms also constitutes a critical component of real-world deployment. Addressing these logistical and organizational challenges will be essential to maximize scalability and long-term effectiveness.

### Limitations

Despite the large sample size used, we can point out some limitations of the study. First, between the second and final data collection, there was a considerable number of dropouts, particularly in the experimental group at the 18-month follow-up. Given the very advanced age of the sample and the high levels of dependency, attrition was frequently related to health deterioration, institutional transitions, or mortality, which are common in long-term studies involving very old populations. Although linear mixed-effects models allow the inclusion of all available data under a missing-at-random assumption, differential attrition between groups may still have introduced some degree of bias. The findings should therefore be interpreted with appropriate caution. Although baseline characteristics did not differ between completers and dropouts, the markedly higher attrition observed in the experimental group compared with the control group remains a potential source of bias and may limit the causal interpretation of long-term effects. Therefore, the 18-month findings should be interpreted with particular caution.

On the other hand, several baseline differences between the control and experimental groups were observed in some outcome measures. Although such imbalances are not uncommon in studies with moderate sample sizes, they represent a potential limitation of the present work. To address this issue, longitudinal analyses were conducted using linear mixed-effects models. These models account for individual baseline variability through subject-specific random intercepts and focus on change over time rather than single post-intervention comparisons. Consequently, the primary conclusions are based on group × time interactions, which are less sensitive to baseline differences than cross-sectional comparisons. Baseline-adjusted mixed-effects models ensure that intervention effects reflect differential change over time rather than cross-sectional differences at baseline, thereby reducing the risk that initial imbalances distort longitudinal inferences. Nevertheless, baseline imbalances should be considered when interpreting the findings, and future studies may benefit from larger samples or stratified randomization procedures.

Another limitation relates to the lack of full blinding and the absence of an active control group. Due to the nature of the intervention, neither participants nor intervention staff could be blinded to group allocation, although outcome assessors remained blinded. In addition, participants in the control group continued their usual activities without receiving an alternative structured intervention. Therefore, some of the observed benefits in the experimental group may partly reflect nonspecific factors such as increased attention, expectancy effects, or greater social engagement. Future studies incorporating active comparison groups would help clarify the specific mechanisms underlying the observed effects.

The heterogeneity of the intervention is another important limitation. Although the program established a predefined number of sessions and intervention structure for each therapeutic profile, this plan was not rigidly applied. From the outset, flexibility was built into each profile to allow adjustments based on individual characteristics and evolving participant needs. The number of sessions assigned to each profile served as a standardized reference point, ensuring a common foundation for intervention and facilitating comparability among participants. However, this number could vary slightly depending on factors such as health status, level of participation, changes in personal or social circumstances, or the emergence of new needs identified during ongoing follow-up. These adjustments were made in a controlled and consensual manner by the multidisciplinary team. The aim was to respond appropriately to each participant’s situation without compromising the overall program design. Whenever possible, the differences within each profile were kept to a minimum, while always maintaining the essential components of the intervention and ensuring comparable exposure to the program’s key elements.

Regarding reproducibility, it is important to emphasize that this was an experimental project involving very old participants. Specifically, 63% were over 80 years old and more than 17% were over 90. In addition, over 76% had a recognized dependency status, and more than 50% were classified as highly dependent (Level II) or severely dependent (Level III). The intervention was delivered in real-world settings, either in participants’ homes or in residential care facilities. Therefore, multiple factors introduce variability and limit the controlled replicability of the experiment.

A further limitation of the study is the potential for selection bias. Participation required a minimum level of cognitive functioning and access to digital technology. Although support was provided when necessary, this requirement may have led to the underrepresentation of individuals with more severe cognitive impairment or limited technological resources. Consequently, the findings may be less generalizable to highly digitally excluded populations, and future research should explore strategies to enhance accessibility and reduce technology-related barriers.

### Future directions

Across the available literature and the findings of the present study, a recurring limitation of multicomponent interventions is the reduced likelihood of observing significant effects when intervention periods are short. Future research should therefore carefully consider intervention duration and include preliminary or pilot studies to determine the minimum exposure needed to achieve meaningful and cost-effective benefits. In addition, incorporating post-intervention follow-up assessments would allow a more precise evaluation of the sustainability and long-term trajectories of observed effects.

Although many multicomponent interventions primarily target physical capacity and cognitive functioning, relatively few are specifically designed to improve instrumental activities of daily living (IADL). Future studies should explicitly focus on IADL outcomes when functional independence is a primary goal. It is also important to acknowledge that commonly used IADL scales may have limited sensitivity to early or subtle changes, particularly in short-term interventions. This may partly explain the lack of detectable transfer effects reported in the literature.

In light of the limited effects observed in balance and gait outcomes, future interventions should also consider hybrid delivery models that combine remote digital sessions with periodic in-person, supervised physical training. Such approaches may allow the scalability and accessibility of telehealth-based programs while ensuring sufficient intensity, progression, and task-specific practice to elicit measurable improvements in mobility-related outcomes. Integrating structured face-to-face components may be particularly relevant when targeting balance, fall prevention, and higher-level functional autonomy.

Evidence from previous reviews suggests that the benefits of ICT-based interventions on social connectedness and social support may be short-lived, often diminishing within six months after the end of the intervention [67]. Consequently, future interventions should incorporate extended follow-up periods to clarify the durability of these effects and to identify the mechanisms through which digital interventions alleviate social isolation and loneliness.

The present study found comparable outcomes among institutionalized participants and those receiving home-based care, particularly in quality of life, perceived loneliness, and depressive symptoms. This finding suggests that such interventions may be adaptable across care contexts; however, future research should employ more homogeneous and well-balanced samples to confirm these observations. Stratification by living arrangement, age, sex, or clinical profile (e.g., cognitive impairment or neurological conditions) would help clarify which subgroups benefit most from the intervention.

Finally, future research should explore the integration of digital assistive technologies and emerging artificial intelligence tools.

The development of customized applications and smart sensors embedded in everyday devices could enable continuous, unobtrusive monitoring of clinically relevant behaviors. These may include physical activity, sleep patterns, and social engagement [68]. These approaches may enhance personalization, improve outcome monitoring, and support more precise evaluation of intervention effectiveness over time.

## Conclusions

This multimodal intervention was associated with favorable longitudinal changes across psychological, social, and functional domains. These effects were assessed using baseline-adjusted linear mixed-effects models with standardized and validated instruments. The findings indicate moderate to very large model-based standardized effects in cognition, depressive symptoms, perceived loneliness, quality of life, and basic functional capacity, suggesting that this approach may contribute to supporting well-being and functional independence in older adults living at home or in residential care settings.

Peer support and interpersonal communication appear to play a relevant role by providing meaningful social interaction, which may contribute to early reductions in depressive symptoms and perceived loneliness. In this study, the use of digital technologies in older adult care was associated with positive psychosocial outcomes. This highlights their potential as a complementary tool within multimodal geriatric interventions.

Notably, many of the observed benefits emerged during the early stages of the intervention and were subsequently maintained over time. This occurred despite a relatively low weekly time commitment, suggesting feasibility in real-world contexts. No meaningful effects were observed in iADL or in balance and gait performance, indicating that physical mobility outcomes may require higher-intensity or hybrid (in-person plus remote) delivery formats to achieve measurable change.

From a public health perspective, the proposed system may represent a scalable and resource-efficient complement to existing healthcare services. Nevertheless, further research is needed to confirm the long-term sustainability, generalizability, and cost-effectiveness of these findings in larger, more diverse populations and under different implementation conditions.

## Supplementary Information

Below is the link to the electronic supplementary material.

Supplementary Material 1

Supplementary Material 2

## Data Availability

The data supporting the findings of this study are not publicly available due to privacy and ethical restrictions to protect participant confidentiality. However, the datasets are available from the corresponding author upon reasonable request.
