# Supplementary material for: A randomized controlled trial to address a multimodal intervention in the elderly: the effects of the CAMINN study
Source: Aging Clin Exp Res. 2026 Mar 25;38(1):125. doi: 10.1007/s40520-026-03371-x (PMC13194253; doi:10.1007/s40520-026-03371-x)
Supplement: Supplementary file 1 — Supplementary Material 1 [file 40520_2026_3371_MOESM1_ESM.docx]

Table S1. Participants’ adherence data.

| Profile | Participants (%) | Planned sessions | Delivered sessions | Execution rate |
| --- | --- | --- | --- | --- |
| Cognitive impairment | 7.5 | 1192 | 980 | 82% |
| Active aging | 20.6 | 2697 | 2449 | 91% |
| Social exclusion | 5 | 412 | 281 | 68% |
| Physical limitation | 8.1 | 756 | 653 | 86% |
| Unwanted loneliness | 15 | 3765 | 3072 | 82% |
| Emotional disorders | 6.3 | 1162 | 935 | 80% |
| Home-based users (total) | 62.5 | 9984 | 8370 | 84% |
| Residential centers | 37.5 | 3412 | 3251 | 95% |
| Total experimental sample | 187 | 13,396 | 11,621 | 87% |

**Activities developed for every single domain of the intervention**

**Cognitive sessions**

Executive Functions, Logical Thinking, and Problem Solving. Evaluates mental agility and the ability to solve sequential and logical problems. Tasks such as:

Word searches about historical places (January, Week 1) - Memory exercises with pairs of images and words (January, Week 2) - "Spot the Difference" game between images (January, Week 4) - Memory game with digital cards (February, Week 2) - Visual attention games with interactive boards (March, Week 3) - Solving simple mazes in digital format (April, Week 1). - "Memorize and repeat" with words displayed for a few seconds (April, Week 4) - Memory games with family photos (May, Week 3) - "Spot the error" activities in images or texts (June, Week 2) - Episodic memory exercises (June, Week 4) - Visual recognition exercises with landscapes (August, Week 1) - Memory games with autumn images (September, Week 1) - Visual attention exercises with patterns and sequences (October, Week 2) - Memory games using images of everyday objects (October, Week 4) - Games to complete Christmas stories with open endings (December, Week 1) - Activity to remember significant events of the year (December, Week 2) - Episodic memory exercises about family celebrations (December, Week 4)

Executive Functions, Logical Thinking, and Problem Solving. Evaluates mental agility and the ability to solve sequential and logical problems. Tasks such as:

Crossword puzzles about everyday objects (January, Week 3) - Solving simple logic problems (February, Week 4) - Number or letter sequence games (April, Week 3) - Nature-related riddles (May, Week 1) - Ordering historical events on a timeline (May, Week 3) - Solving basic math puzzles (June, Week 3) - Solving visual problems such as "Find the correct path" (July, Week 2) - Ordering fictional events on a timeline (August, Week 2) - Riddles with verbal or visual clues (August, Week 4) - Solving simple math puzzles (September, Week 2) - Solving small logic problems related to everyday life (October, Week 3) - Solving crossword puzzles with food-related words (November, Week 2) - Solving visual puzzles with winter themes (December, Week 3)

Expression and Language. Evaluates verbal fluency, association of ideas, and the ability to structure sentences and narratives. Activities such as: completing familiar sentences or proverbs (March, Week 2) - Guessing the meaning of unusual words (March, Week 4) - Fill-in-the-blank word games (May, Week 2) - "Complete the sentence" games with travel themes (June, Week 1) - Group short story creation activity (July, Week 3) - Group story creation exercise with autumn themes (September, Week 4) - Sentence completion games about healthy habits (November, Week 2) - List-making activity on everyday topics (November, Week 4)

Association and Classification. Assesses the ability to categorize items and establish connections between them. Activities such as: Categorization games (colors, fruits, objects) (February, Week 3) - Word associations related to home (February, Week 1) - Ordering sequences of narrative images (March, Week 1) - Classifying images by category (animals, food, objects) (April, Week 2) - Visual associations between images and names (Older, Week 4) - Word association game related to summer (July, Week 1) - Classifying images of summer fruits and flowers (July, Week 4) - Associating popular songs with personal memories (August, Week 3) - Word association activities related to the season (September, Week 3) - Categorization activities with autumn elements (October, Week 1) - Exercises associating images with historical concepts (November, Week 1)

**Physical activity sessions**

The physical activity sessions were designed for small groups and adapted to each participant's functional abilities, always ensuring safety. Activities included joint mobility exercises, upper and lower limb muscle strengthening, balance and coordination exercises aimed at fall prevention, as well as breathing and body awareness exercises. In some cases, functional rehabilitation exercises were incorporated, especially for participants who had recently undergone surgery or had mild physical limitations. Sessions were conducted both seated and standing, depending on the participant's ability, and always with clear instructions and visual demonstrations from the professional.

**Recreational sessions**

Activities to cover the different subscales of the FUMAT scale such as: Valentine's Day (February 14) o Classic Cinema (February) o International Women's Day (March 8) o Holy Week (April) o Musical Bingo (April, week 2) o Healthy Cooking Workshop (April) o Memory Game with Family Photos (May, week 3) o Summer Party (June) o Music and Movement Week (July) o "Guess the Song" (September, week 4) o Short Story Workshop (October) o Laughter Therapy Session (November, week 4), Healthy Eating Workshop (November) • "Guess the Christmas Carol" Activity (December, week 3), etc.

**Individual follow-up session with social worker/ psychologist**

The weekly follow-up sessions with the social worker focused primarily on providing ongoing psychosocial support. These sessions addressed aspects related to the user's overall well-being, potential changes in their family or social environment, identifying vulnerabilities, reinforcing their sense of security, and promoting personal autonomy. They also included reviewing program adherence, working on motivation, and, when necessary, coordinating with family members or other community resources. The follow-up sessions with the psychologist centered on emotional support and psychological well-being.
