# Supplementary figures and images for: A randomized controlled trial to address a multimodal intervention in the elderly: the effects of the CAMINN study
Source: Aging Clin Exp Res. 2026 Mar 25;38(1):125. doi: 10.1007/s40520-026-03371-x (PMC13194253; doi:10.1007/s40520-026-03371-x)

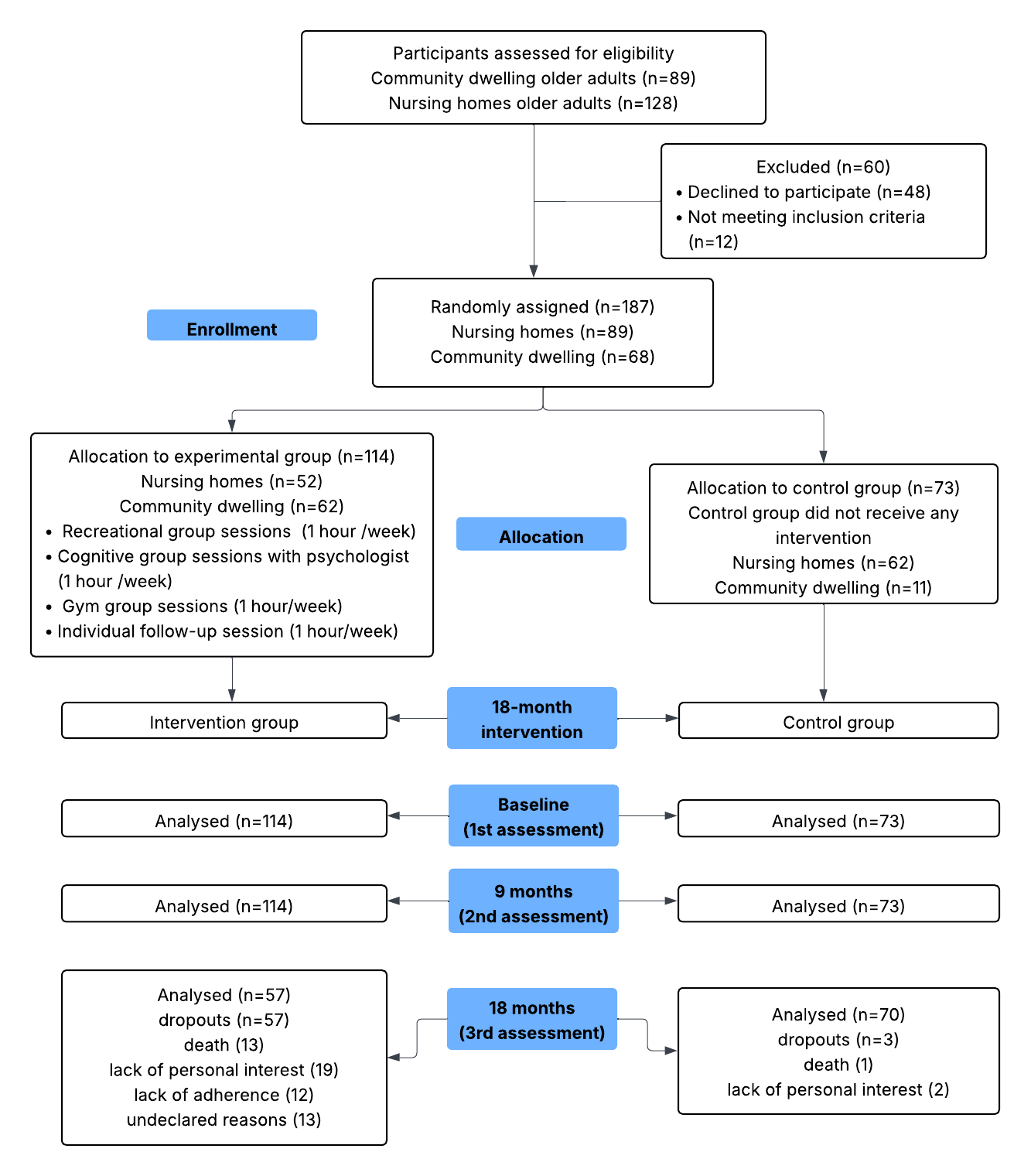

Supplement: Supplementary file 2 — Supplementary Material 2 [file 40520_2026_3371_MOESM2_ESM.tiff]
